# Transcriptional profiling of equine endometrium before, during and after capsule disintegration during normal pregnancy and after oxytocin-induced luteostasis in non-pregnant mares

**DOI:** 10.1371/journal.pone.0257161

**Published:** 2021-10-06

**Authors:** Claudia Klein, Phoebe Bruce, Jutta Hammermueller, Tony Hayes, Brandon Lillie, Keith Betteridge

**Affiliations:** 1 Friedrich-Loeffler-Institute, Institute of Farm Animal Genetics, Mariensee, Germany; 2 Department of Pathobiology, Ontario Veterinary College, University of Guelph, Guelph, Ontario, Canada; 3 Department of Biomedical Sciences, Ontario Veterinary College, University of Guelph, Guelph, Ontario, Canada; University of Florida, UNITED STATES

## Abstract

The current study used RNA sequencing to determine transcriptional profiles of equine endometrium collected 14, 22, and 28 days after ovulation from pregnant mares. In addition, the transcriptomes of endometrial samples obtained 20 days after ovulation from pregnant mares, and from non-pregnant mares which displayed and failed to display extended luteal function following the administration of oxytocin, were determined and compared in order to delineate genes whose expressions depend on the presence of the conceptus as opposed to elevated progesterone alone. A mere fifty-five transcripts were differentially expressed between samples collected from mares at Day 22 and Day 28 of pregnancy. This likely reflects the longer-term exposure to a relatively constant, progesterone-dominated environment with little change in factors secreted by the conceptus that would affect endometrial gene expression. The complement system was amongst the canonical pathways significantly enriched in transcripts differentially expressed between Day 14 and Day 22/28 of pregnancy. The expression of complement components 7 and 8 was confirmed using in situ hybridization. The expression of SERPING1, an inhibitor of the complement system, was confirmed by immunohistochemistry. In line with the resumed capacity of the endometrium to produce prostaglandin, prostaglandin G/H synthase 1 was expressed at higher levels at Days 22 and 28 than at Day 14 of pregnancy. Our data suggest that this up-regulation is enhanced by the presence of the conceptus; samples obtained from mares at Day 20 of pregnancy had significantly higher levels of prostaglandin G/H synthase 1 transcript than mares with extended luteal function.

## Introduction

As in other domestic species [1], pregnancy maintenance in the mare depends upon progestogens secreted by the corpus luteum (CL) and efficient biochemical signaling between the conceptus and the mother, collectively referred to as “maternal recognition of pregnancy” (MRP) [2]. These processes are best understood in domestic ruminants and other ungulates, notably the pig [3, 4]. The horse is one of the few domestic species in which neither a defined conceptus-derived pregnancy recognition signal, nor a specific stage of pregnancy for its putative production, has been identified. Horses exhibit some unusual features during early pregnancy that probably contribute to pregnancy recognition. For example, the spherical equine conceptus migrates continuously throughout the uterine lumen between Days 9 and 16 after ovulation [5] and restriction of conceptus movement results in luteolysis, with subsequent failure of pregnancy [6]. The necessity of embryonic mobility for establishment of pregnancy has recently been questioned as the insertion of a plastic ball into the uterine lumen of cycling mares prolonged luteal function in 9 of 12 treated mares [7] and in view of the observation that embryo movement ceased in recipients 10 days after ovulation when transferring 10 day old embryos to asynchronous recipient mares [8]. Equine conceptuses produce substantial amounts of estrogens and prostaglandins [9–11] and the bilaminar trophoblast (BT) and trilaminar trophoblast (TT) exhibit functional differences with regard to metabolism of estrogens [12] and prostaglandin production [13]. Another special feature of this process in horses is the transitory appearance of a mucin-like glycoprotein capsule around the blastocyst as it expands in the uterus during the second and third weeks of gestation [14], which is essential to embryo survival during early pregnancy [15]. For the first two-thirds of its existence, the capsule is resilient and favors extensive migration of the spherical conceptus within the uterus until, at about Day 16–17, migration ceases and the conceptus becomes “fixed” at the site of subsequent placentation. Coincidently with fixation, the conceptus becomes flaccid and the capsule changes in composition, with a reduction in its sialic acid content [16, 17]. By Day 22, the capsule no longer envelops the yolk sac [18].

MRP is of an antiluteolytic nature in the horse, in other words, release of Prostaglandin F2alpha (PGF2alpha) from the uterus is prevented, resulting in the absence of episodic peaks of PGF2alpha in the peripheral circulation around the time of expected luteolysis [19]. Likewise, uterine venous plasma [20] and uterine flushings [21] contain less PGF2alpha in pregnant than in non-pregnant mares. Reduced production of PGF2alpha is likely the consequence of reduced expression of prostaglandin-endoperoxide synthase 2, PTGS2, a key enzyme in the production of prostaglandins [22]. Reduced expression of PTGS2 is directly attributable to a thus-far unidentified component of conceptus secretions [22].

Oxytocin is integral to luteolysis in the mare, reflected through its induction of an immediate rise in a PGF2alpha metabolite when administered during late diestrus [23] and the observation that repeated oxytocin administration starting in early diestrus delays luteolysis [24]. Oxytocin responsiveness is altered during early pregnancy in mares, primarily through a reduced binding capacity of the endometrium for oxytocin [25], which in turn is reflected in the reduced expression of the oxytocin receptor protein in pregnant mares [26]. Interestingly, in the mare expression of the oxytocin receptor (OXTR) appears to be regulated at the post-transcriptional level; transcript levels are unaltered by pregnancy status [27, 28] in the face of reduced expression of the protein [26].

Recent advancement in understanding MRP in the mare through studies employing transcriptional and proteomic approaches [27–30] have focused on the first 16 days of pregnancy. According to the Report of the Havemeyer Foundation Workshop on Equine Implantation [31], “The biology of early equine pregnancy is an area in which there is much still to be discovered.” The aim of the current study was therefore to use RNA sequencing to determine transcriptional profiles of equine endometrium collected 14, 22, and 28 days after ovulation from pregnant mares. In addition, the transcriptomes of endometrial samples obtained 20 days after ovulation from pregnant mares, and from non-pregnant mares which displayed and failed to display extended luteal function following the administration of oxytocin, were determined and compared in order to delineate genes whose expressions depend on the presence of the conceptus as opposed to elevated progesterone alone. Selected transcriptomes of interest were further investigated using immunohistochemical and in situ hybridization techniques.

## Materials and methods

### Collection of samples

The mares used to provide endometrial samples were maintained in an experimental herd at the Arkell research station of the Ontario Ministry of Agriculture and Rural Affairs principally for studies of early equine pregnancy under conditions approved by the University of Guelph’s Animal Care Committee [18, 32]. General aspects of the mares’ breeds and reproductive management have been described previously[32]; details of special relevance to the present study are that the developmental stages of samples (relative to the time of ovulation) were known to within ± 0.5 days and that samples of endometrium were retrieved by transcervical biopsy using a pulling biopsy punch (Lane Medical Co., Boulder, CO, USA) as described by Kenney [33]. Immediately after collection, samples were each divided into three pieces, one was fixed for histology while two were transferred to individual dry cryovials, flash-frozen in liquid nitrogen and stored at -80°C pending isolation of RNA.

For transcriptional profiling during normal pregnancy samples distributed over Days 14, 20, 22 and 28 of normal pregnancy were used. To obtain day-20 endometrium from non-pregnant mares with extended luteal function (so that transcriptomes of pregnant and non-pregnant mares at Day 20 could be compared), 13 mares which had not been inseminated were treated between Days 7 and 14 by daily intramuscular injections of 60 IU oxytocin in the expectation that the luteal phase of their estrous cycles would be extended in 60–70% of cases [34]. Daily jugular blood samples, drawn, processed and assayed as previously described [32] were used to distinguish retrospectively between mares in which the luteal phase had or had not been extended up to the time of collection of endometrial samples (8/13 oxytocin-treated mares had extended cycles, 5/13 did not). Extension of luteal phase was determined based on the plasma progesterone values being greater than 2 ng/ml.

### Isolation of RNA

Endometrial RNA was isolated from pregnant mares (Days 14 (n = 4), 20 (n = 8), 22 (n = 5), and 28 (n = 4)), and from mares with extended luteal function (n = 6) and mares that failed to extend luteal function (n = 3) following the repeated administration of oxytocin. Total RNA was isolated from endometrial samples using the RNEasy Mini kit as per manufacturer’s instructions, including sample homogenization in buffer RLT (Qiagen, Redwood City, CA, USA). DNA quantity was assessed by Nanodrop, as well as a Bioanalyzer at the sequencing center.

### Next generation sequencing

Total RNA samples were submitted for mRNA-Seq at the Donnelly Sequencing Center at the University of Toronto. Total RNA was treated with the DNA-free DNA Removal Kit (Thermo Fisher Scientific Inc., Waltham, USA) to remove contaminant DNA. DNase-treated total RNA was then quantified using Qubit RNA BR (Thermo Fisher Scientific Inc., Waltham, USA) fluorescent chemistry and 1 ng was used to obtain RNA Integrity Number (RIN) using the Bioanalyzer RNA 6000 Pico kit (Agilent Technologies Inc., Santa Clara, USA). All samples had a RIN > 8 except two samples (RIN = 7.8); the median RIN score was 8.9. Samples (1 μg) were then processed using the TruSeq Stranded mRNA Library Prep Kit (Illumina Inc., San Diego, USA; protocol v. Document # 1000000040498 v00) including PolyA selection, with 8 minutes of fragmentation at 94 °C and 11 cycles of amplification. One microliter top stock of each purified final library was run on an Agilent Bioanalyzer dsDNA High Sensitivity chip (Agilent Technologies Inc., Santa Clara, USA). The average library size was 294 nt. The libraries were quantified using the KAPA Universal qPCR Master Mix (KAPA Biosystems/Roche) and were pooled at equimolar ratios after size-adjustment. The final pool was run on an Agilent Bioanalyzer dsDNA High Sensitivity chip and quantified using NEBNext Library Quant Kit for Illumina (New England Biolabs, Ipswich, USA). The quantified pool was hybridized at a final concentration of 2.2 pM and sequenced single-end on the Illumina NextSeq 500 platform using two High-Output v2.5 flowcells at 75 bp read lengths.

### Analysis of data

The resulting FASTQ files were submitted for quality control and the ‘trim galore’ function (local Galaxy platform) was used to trim adapter sequences and to remove low quality ends and reads shorter than 50 bp. Reads were then mapped to the equine EquCab3 genome sequence using JMP Genomics 8.2 (SAS, Cary, NC, USA), allowing two mismatches per read. Total counts and transcripts per million (TPM) were generated for all transcripts. Data for Days 14, 22, and 28 of pregnancy and for samples collected 20 days after ovulation from pregnant and oxytocin treated mares were analyzed separately. Only transcripts with at least 10 reads present in all biological replicates for a given treatment were considered present and included in further analyses. Distribution analysis and Correlation and Principal Variance Component Analysis were applied, followed by Spearman’s correlation of all transcripts considered present. Differential gene expression of transcripts considered expressed was modeled using analysis of variance. All analyses were carried out using JMP Genomics 8.2. Transcripts displaying a fold change of 2 or greater and a FDR-adjusted p-value (q-value) of 0.01 or smaller were considered differentially expressed.

Ingenuity Pathway Analysis (Qiagen) was used to identify canonical pathways and upstream regulators. Given that the number of genes differentially expressed between samples collected at Day 22 and Day 28 of pregnancy was small, Ingenuity Pathway Analysis was carried out for Day 22 and Day 28 samples combined in comparison to samples collected at Day 14 of pregnancy. Information on differentially expressed genes was supplemented with additional knowledge from the literature (http://www.ncbi.nlm.nih.gov/PubMed/). Results were furthermore compared to a meta-analysis which determined endometrial receptivity-associated genes of human endometrium [35].

### Real-time PCR analysis

Endometrial RNA was used to prepare cDNA using the QIAGEN QuantiTect Reverse Transcription Kit as per manufacturer’s instructions. Real-time RT-qPCR was then performed on a Roche LightCycler 480 using the LightCycler FastStart DNA MasterPLUS SYBR Green 1 (Roche, Laval, PQ, Canada) according to the manufacturer’s recommendations with primers as listed in S4 Table and annealing temperatures either 60 °C or 64 °C. For all genes, an initial 5 min preincubation at 95 °C was followed by 45 cycles of denaturation (95 °C for 10 s), annealing for 10 s and elongation (72 °C for 15 s) and detection. Standard curves using pooled cDNA were performed, PCR products were sequenced to confirm identity, and melting-curve analysis verified amplification of a single produce. All samples were analyzed in duplicate, and the mean expression ratios (target gene:reference gene expression) using *GAPDH* as the reference gene were determined by RelQuant software from Roche Diagnostics.

### Immunohistochemistry

Tissues were embedded in paraffin by immersing tissue in a series of ethanol solutions of increasing concentrations until 100%, water-free alcohol was reached. Following dehydration, the tissue was immersed in xylene, followed by embedding in paraffin. Sections were cut at 5 μm and mounted onto positively charged slides. Immunohistochemistry (IHC) was performed on an automated IHC stainer, BOND-MAX (Leica, Richmond Hill, ON, Canada), as described [36]. A polyclonal rabbit anti-human SERPING1 antibody (NBP2-14892, Novus Biologicals Littleton, Colorado, USA) and samples obtained from mares at Day 14, Day 22 and Day 28 of pregnancy were used. As negative control, primary antibody was replaced with rabbit normal serum.

### *In situ* hybridization

In situ hybridization using custom designed probes targeting equine complement component 7 (C7) and equine complement component 8 (C8) was carried out using the RNAScope^®^ system (Advanced Cell Diagnostics, Inc., Newark, CA). The manufacturer’s instructions for the RNAscope 2.5 HD Assay—BROWN were followed. Following deparaffinization, samples were exposed to hydrogen peroxide for 10 min followed by a wash step using water. Slides were then submerged into boiling RNAscope^®^ Target Retrieval Reagents for 15 min. Following target retrieval, samples were exposed to RNAscope^®^ Protease Plus for 30 min at 40 °C. The probe was hybridized for 2 h at 40 °C after which signal detection was carried out as described in the manufacturer’s instruction, with staining presenting as brown punctate dots. A negative control consisting of a scrambled probe was included.

## Results

### Analysis of data

**Fig 1:**
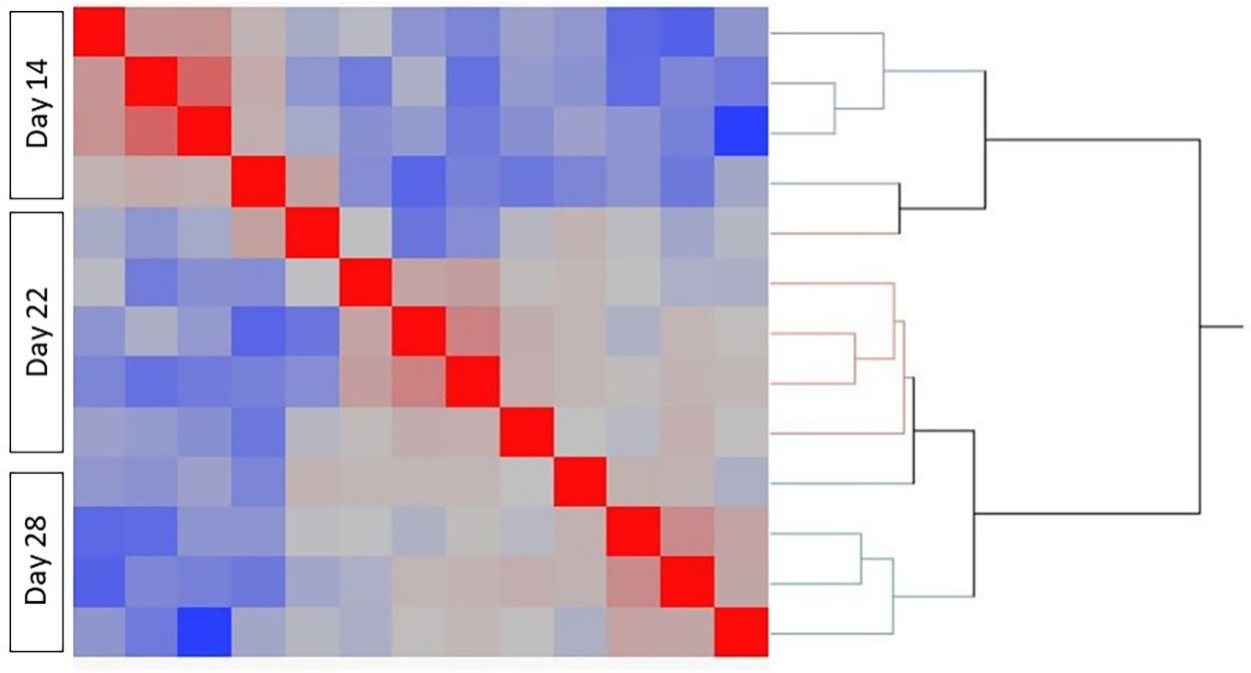
Comparison of transcriptional profiles across samples collected at Day 14, 22, and 28 of pregnancy. Heatmap visualizing the hierarchically clustered Spearman correlation matrix.

**Fig 2:**
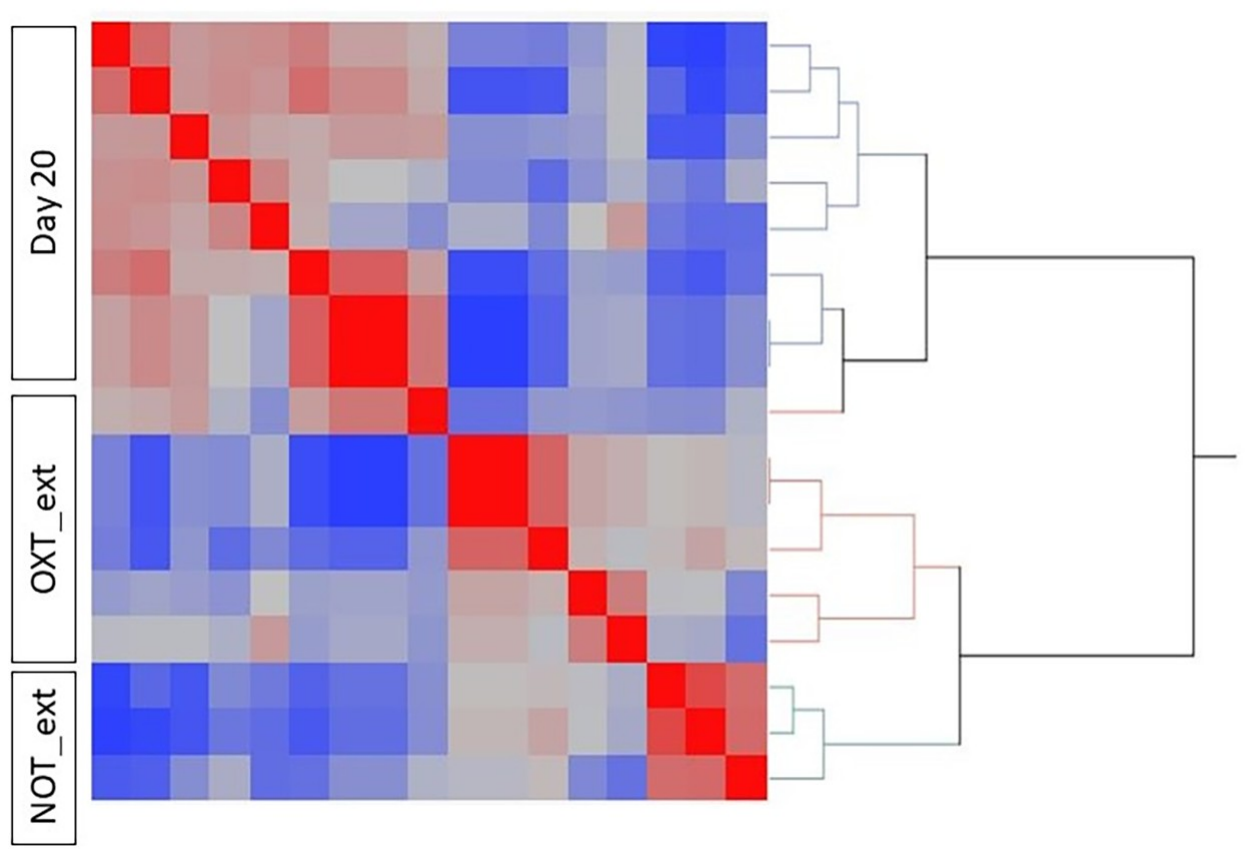
Comparison of transcriptional profiles across samples collected 20 days after ovulation from pregnant mares (Day 20), and from mares which displayed (OXT_ext) and failed to display (NOT_ext) extended luteal function following the administration of oxytocin. Heatmap visualizing the hierarchically clustered Spearman correlation matrix.

The number of aligned reads averaged 29,572,135 ± 4,838,259 (raw and processed data are available in the Gene Expression Omnibus (GEO) database, accession number GSE180022). A total of 15,697 transcripts were expressed across all treatment groups. Figs 1 and 2 show the Spearman’s correlation heatmap of expressed TPM values. When clustering Days 14, 22, and 28 of pregnancy (Fig 1), Day 14 samples are clustering separately from Days 22 and 28. Likewise, samples collected at Day 20 of pregnancy cluster separately from samples collected from mares with extended and failed extension of the luteal phase following the administration of oxytocin (Fig 2).

**Fig 3:**
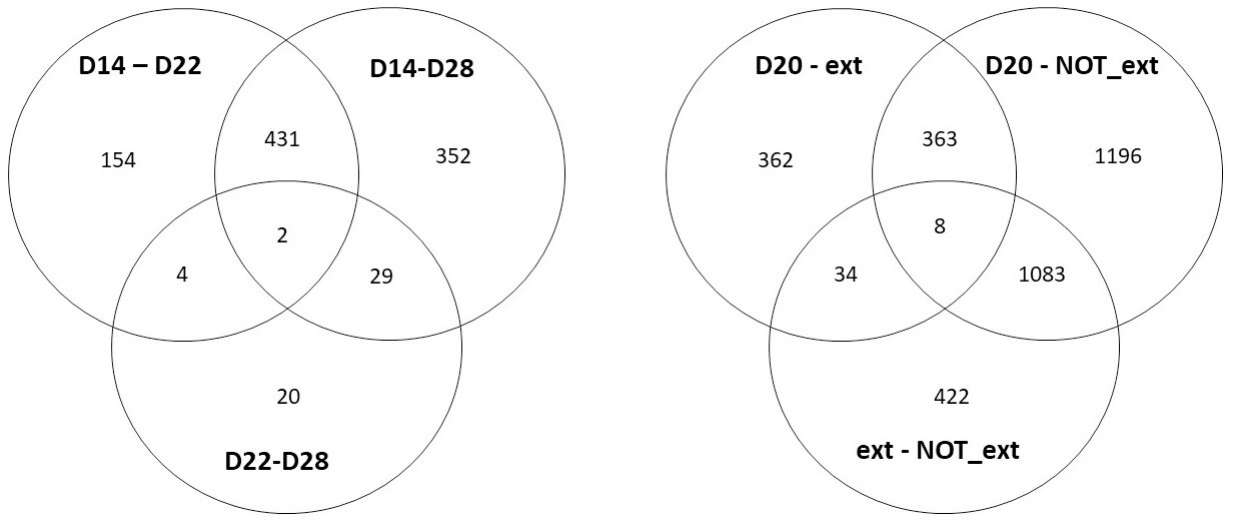
Venn diagram of transcripts differentially expressed in endometrial samples collected from mares at Days 14, 22, and 28 of pregnancy (left) and samples obtained 20 days after ovulation from pregnant mares, and from mares which displayed and failed to display extended luteal function following the administration of oxytocin. D14, D20, D22, D28 = Day 14, 20, 22, 28 of pregnancy; ext = extended luteal function following the administration of oxytocin; NOT_ext = failed luteal extension following the administration of oxytocin.

As shown in Fig 3, 992 transcripts were differentially expressed between samples collected at Days 14, 22, and 28 of pregnancy: most differential expression was found between pre-fixation and post-fixation (Day 14 and Day 22, and Day 14 and 28 of pregnancy; 591 and 814 transcripts respectively), while only 55 transcripts were differentially expressed between Day 22 and Day 28 of pregnancy. S1 Table lists TPM values, p-values, and fold changes for all differentially expressed transcripts between samples collected at Days 14, 22, and 28 of pregnancy. Comparison of samples collected at Day 20 of pregnancy and from mares that displayed and failed to display extended luteal function following the administration of oxytocin revealed 3472 transcripts that were differentially expressed (Fig 3). Most of these (2650) were differentially expressed between Day 20 of pregnancy and mares that failed to display extended luteal function. Comparison of mares with extended luteal function versus mares that failed to extend their luteal function following the administration of oxytocin revealed 1547 differentially expressed transcripts. Between mares at Day 20 of pregnancy and mares with oxytocin-extended luteal function 767 transcripts were differentially expressed. S2 Table lists TPM values, p-values, and fold changes for all transcripts differentially expressed between Day 20 of pregnancy and from mares that displayed and failed to display extended luteal function following the administration of oxytocin.

**Fig 4:**
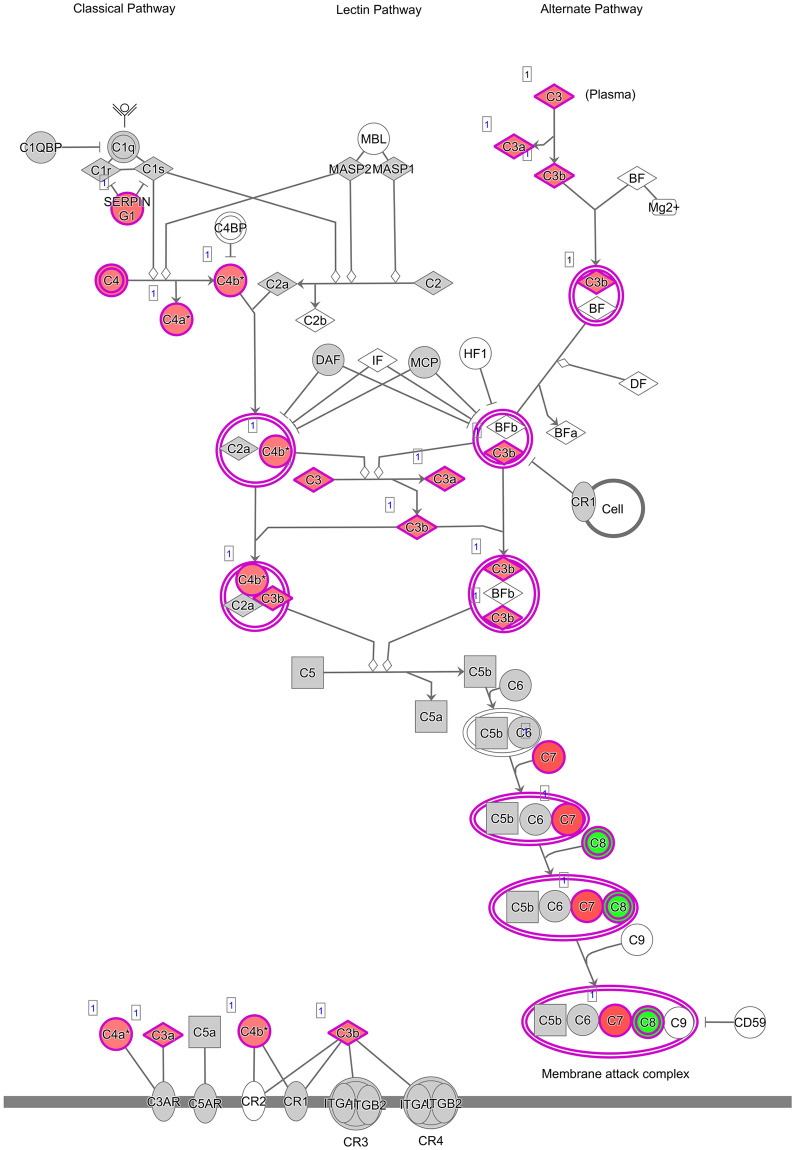
IPA representation of the complement system pathway enriched during normal pregnancy (Day 14 versus Day 22 & 28). Grey symbols: transcripts are expressed but not differentially regulated. Red symbols: transcripts are expressed at higher levels at Day 14 of pregnancy. Green symbols: transcripts are expressed at lower levels at Day 14 of pregnancy).

Canonical pathways identified in the course of Ingenuity Pathway Analysis are depicted in S1 Fig. The canonical pathway “complement system” which is displayed in detail in Fig 4, was investigated in further detail through immunohistochemical analysis of SERPING1 and through in situ hybridization of *C8G* and *C7*.

Results were furthermore compared to a meta-analysis which determined endometrial receptivity-associated genes of human endometrium [35]. Of the 57 genes identified as markers of endometrial receptivity in this meta-analysis, 13 were differentially expressed in the present study, 30 were expressed but not differentially regulated, 4 genes did not meet the classification criterion of being considered expressed, and 10 genes were not present in the annotation used to align the sequencing data (S3 Table).

### Real-time PCR analysis

**Fig 5:**
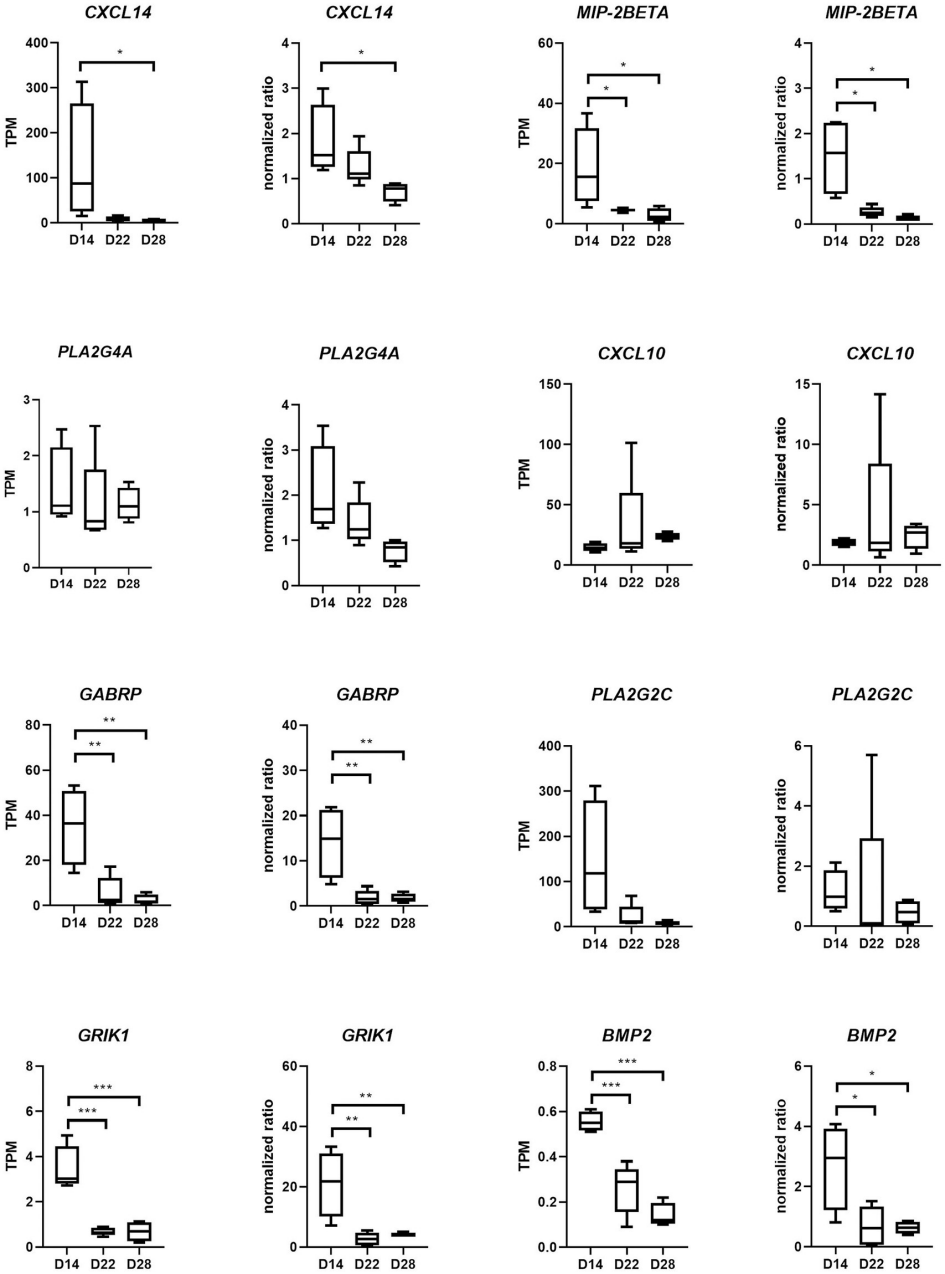
Real-time PCR results of selected transcripts expressed in endometrial samples collected from mares at Days 14, 22, and 28 of pregnancy. TPM values (RNA seq) and normalized ratios (qPCR) for each transcript are shown side-by-side. *: P ≤ 0.05, **: P ≤ 0.01, ***: P ≤ 0.001.

**Fig 6:**
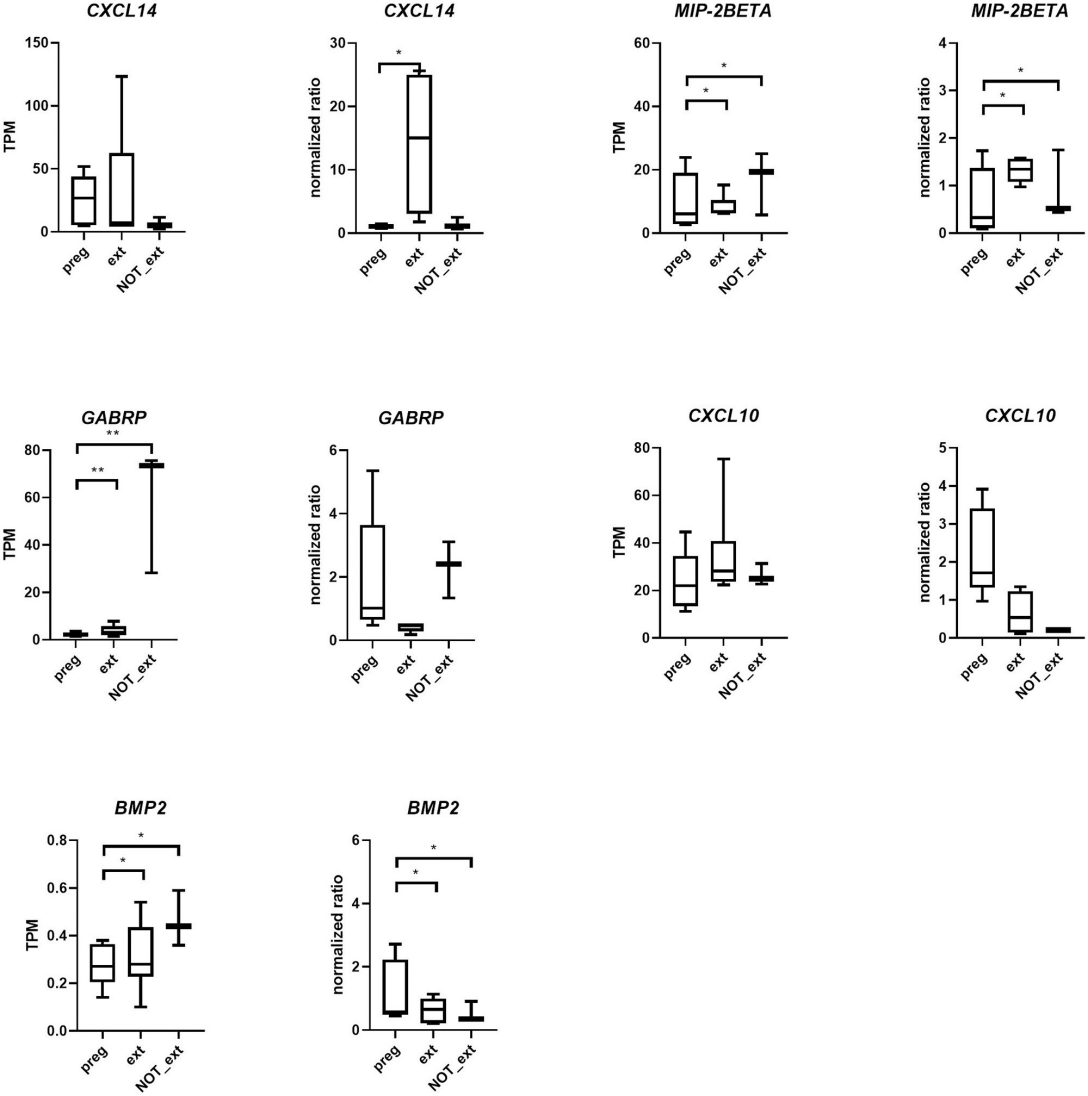
Real-time PCR results of selected transcripts expressed endometrial samples obtained 20 days after ovulation from pregnant mares, and from mares which displayed and failed to display extended luteal function following the administration of oxytocin. TPM values (RNA seq) and normalized ratios (qPCR) for each transcript are shown side-by-side. *: P ≤ 0.05, **: P ≤ 0.01.

Real-time PCR analysis was used to verify selected expression data obtained from the RNA sequencing analysis. For selected transcripts expressed in endometrial samples collected from mares at Days 14, 22, and 28 of pregnancy, expression data obtained by real-time PCR analysis paralleled those obtained by RNA sequencing analysis for all 8 transcripts analyzed (Fig 5). For selected transcripts expressed in endometrial samples obtained 20 days after ovulation from pregnant mares, and from mares which displayed and failed to display extended luteal function following the administration of oxytocin., expression data obtained by real-time PCR analysis paralleled those obtained by RNA sequencing analysis for 3 of 5 transcripts analyzed (Fig 6).

### Immunohistochemistry

**Fig 7:**
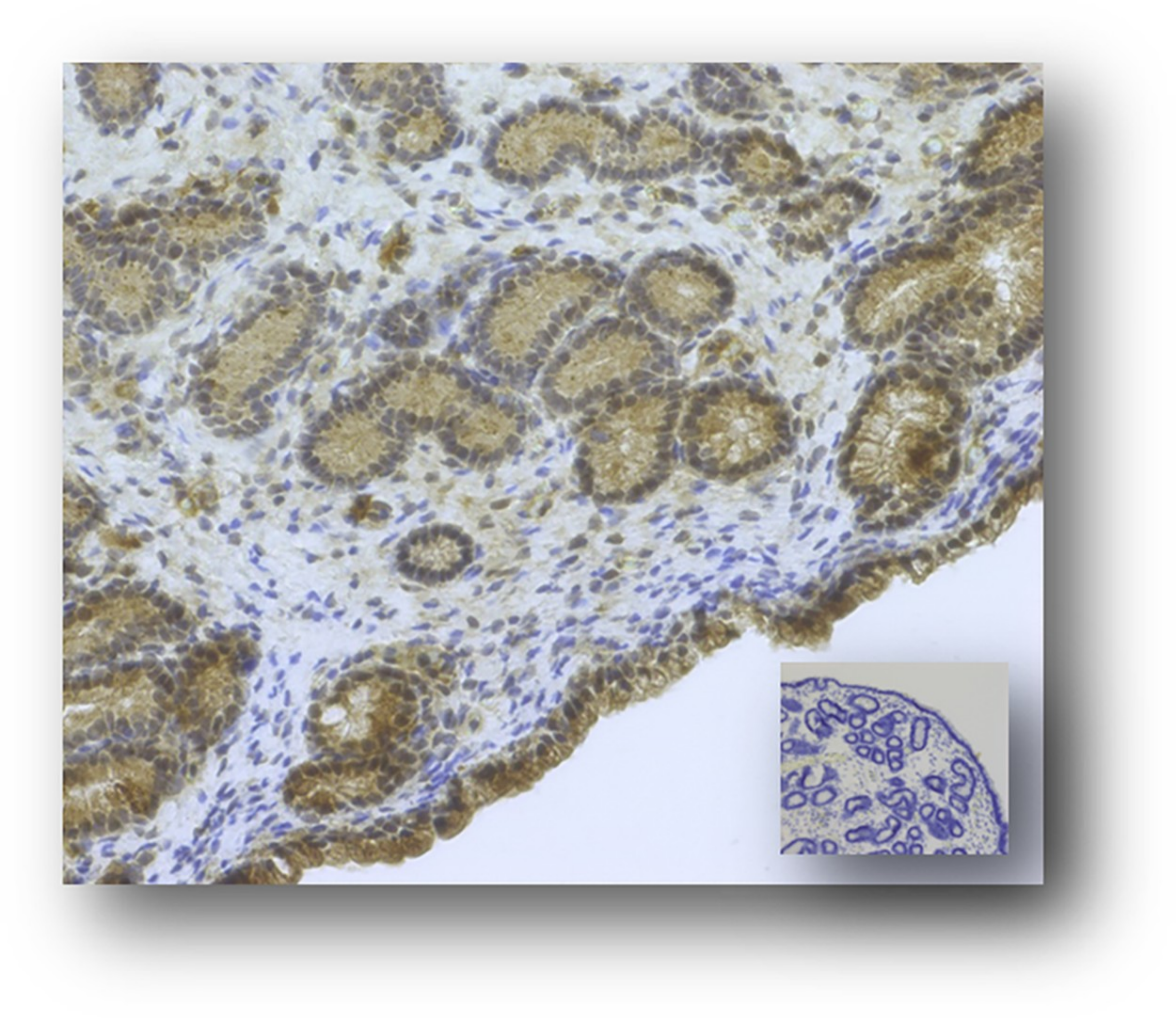
Immunohistochemical localization of SERPING1 expression in equine endometrium at Day 28 of pregnancy. Inserted image shows the negative control. All images are shown at 200× magnification. Size bar (50 μm) for reference; *: luminal epithelium; →: glandular epithelium.

Immunohistochemistry was used to locate SERPING1 protein expression within the equine endometrium during early pregnancy. A polyclonal rabbit antibody raised against human SERPING1 was confirmed to recognize the equine protein based on a prominent band of approximately 55 kDa upon Western Blot analysis. Protein expression was strongest in luminal epithelial cells and slightly less pronounced in glandular epithelial cells. Stromal cells displayed weak staining intensity. No obvious difference in staining intensity were visible between samples collected at Day 14, Day 22 or Day 28 of pregnancy (representative image of a Day 28 sample shown in Fig 7). No staining was visible upon replacement of the primary antibody with rabbit normal serum (Fig 7).

### *In situ* hybridization

**Fig 8:**
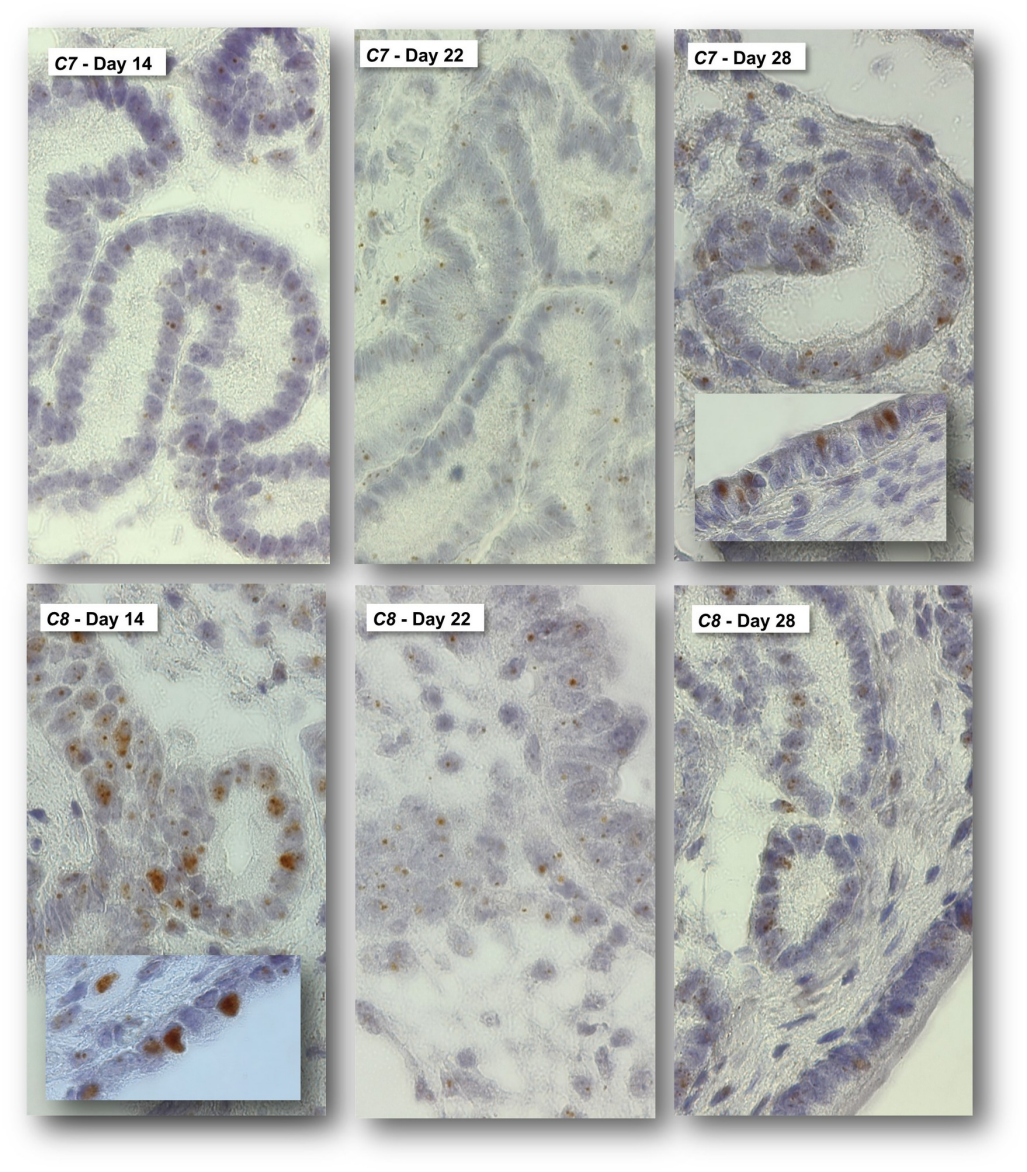
*In situ* hybridization analysis of complement component 7 (*C7*) and equine complement component 8 (*C8*) in equine endometrium at Day 14, 22, and 28 of pregnancy. Staining presents as brown dots. Inserted images show staining of individual luminal epithelial cells present in at Day 28 (*C7*) and Day 14 (*C8*). All images are shown at 630× magnification. Size bar (10 μm) for reference. LE: luminal epithelium.

Custom designed probes were used to localize the mRNA of equine complement component 7 (*C7*) 8 (*C8*) in equine endometrium at Day 14, 22, and 28 of pregnancy (Fig 8). Both *C7* and *C8* were predominantly expressed by glandular epithelial cells (brown punctuated dots). *C7* was expressed at lower levels than *C8* which is in accordance with the TPM values observed for *C7* and *C8*. On Day 28 of pregnancy, individual luminal epithelial cells also stained positive for *C7*. Likewise, individual luminal epithelial cells displayed strong staining intensity for *C8* at Day 14 of pregnancy.

## Discussion

In this report we present the transcriptome analysis of equine endometrium during normal pregnancy and, in non-pregnant mares, during luteal phases extended (or not) by oxytocin treatment. We report the importance of the complement component cascade and, for the first time, distinguish between the gene expression patterns ascribable to progesterone alone and those that are dependent on the presence of a conceptus. Time points for using RNA sequencing to determine transcriptional profiles of equine endometrium during pregnancy were chosen to include endometrial samples during the presence of a conceptus (i) surrounded by an intact capsule (Day 14), (ii) near the time of capsule disintegration (Day 22), and (iii) without a capsule (Day 28). In addition, we compared the transcriptional profiles of endometrial samples obtained 20 days after ovulation from pregnant mares, and from mares which either displayed or failed to display extended luteal function following the administration of oxytocin. The latter comparison was included to delineate genes whose expression is driven by progesterone as opposed to conceptus’ secretions. A caveat is the unknown extent to which repeated administration of oxytocin itself might have induced changes in gene expression. Given that the last dose of oxytocin was administered 6 days before collection of samples, we would expect the effect of oxytocin on the transcriptome to be minimal.

A mere fifty-five transcripts were differentially expressed between samples collected from mares at Day 22 and Day 28 of pregnancy. This likely reflects the longer-term exposure to a relatively constant, progesterone-dominated environment with little change in factors secreted by the conceptus that would affect endometrial gene expression. Contrarily, more transcripts were found to be differentially expressed when comparing Day 14 to Days 22 and 28 of pregnancy, indicating that the endometrium undergoes major transcriptional changes between the second and third weeks of pregnancy.

A puzzling aspect of early pregnancy in the horse is the transient nature of the reduction of prostaglandin production by the endometrium. Uterine flushings obtained from mares beyond Day 18 of pregnancy contain concentrations of PGF2alpha similar to those seen during the time of luteolysis in mares [13] and by Day 18 of pregnancy the uterus has resumed responsiveness to oxytocin [25]. In line with the resumed capacity of the endometrium to produce prostaglandin, prostaglandin G/H synthase 1 (*PTGS1*) was expressed at higher levels at Days 22 and 28 than at Day 14 of pregnancy. Atli and co-workers reported similar findings and described higher expression of *PTGS1* at Days 18 and 22 of pregnancy compared to Day 15 of pregnancy [37]. Our data suggest that *PTGS1* up-regulation is enhanced by the presence of the conceptus; samples obtained from mares at Day 20 of pregnancy had significantly higher levels of *PTGS1* transcript than mares with extended luteal function. Similar to *PTGS1*, 15-hydroxyprostaglandin dehydrogenase (*HPGD*) displayed higher expression at Day 28 than at Days 14 and 22 of pregnancy. Atli and co-workers had previously reported an up-regulation of HPGD at Day 22 of pregnancy compared to Day 18 of pregnancy [37]. HPGD is a rate-limited prostaglandin-degrading enzyme [38], which is likely to counteract the increased capacity of the endometrium to produce prostaglandin. Up-regulation of *HPGD*, like that of *PTGS1*, is a pregnancy-specific event, as mare with extended luteal function displayed lower *HPGD* abundance than did mares at Day 20 of pregnancy. The prostaglandin receptor (*PTGFR*) was expressed at lower levels at Days 22 and 28 of pregnancy than earlier, likely presenting another mechanism by which the endometrium protects itself from the resumed capacity of prostaglandin production. Mares at Day 20 of pregnancy and mares that underwent oxytocin-induced luteostasis expressed lower levels of *PTGFR* than mares that failed to undergo luteostasis, indicating that the down-regulation of *PTGFR* is unlikely to be pregnancy-specific.

Uterocalin (*P19*) was the most abundant transcript among progesterone-stimulated transcripts, i.e. transcripts with higher expression during extended luteal function and at Day 20 of pregnancy. During pregnancy we observed a down-regulation of P19, which agrees with previous reports [39, 40]. Similar to P19, lipocalin 1 (*LCN1*) was down-regulated at Day 22 and Day 28 when compared to Day 14 of pregnancy. P19 was the only progesterone-dependent transcript which displayed a down-regulation during pregnancy. Other progesterone-stimulated transcripts such as uteroferrin (*ACP5*), lipocalin 2 (*LCN2*), and epidermal growth factor (*EGF*) were stably expressed during Days 14, 22, and 28 of pregnancy. Stanniocalcin 1 (*STC1*), evidently another progesterone-regulated transcript based on its up-regulated expression in endometrium of pregnant mares and mares with extended luteal function, displayed higher expression at Day 28 than at Day 14 of pregnancy. Increased expression during pregnancy, and induction of *STC1* by progesterone in ovariectomized females have previously been reported for the sheep and pig [41, 42]. *PGRMC1* was expressed at higher levels in mares that failed that to undergo luteal maintenance following the administration of oxytocin and was expressed at lower levels at Day 22 and 28 of pregnancy, likely indicating a progesterone-dependent down-regulation.

The relaxin family peptide receptor 1 (*RXFP1*) underwent a pregnancy-specific up-regulation—its transcript levels were higher at Day 20 of pregnancy than in mares that underwent luteostasis. We have previously described the expression of relaxin by the pre-implantation equine conceptus [43], and the pregnancy-specific up-regulation of *RXFP1* underlines relaxin’s potential role in angiogenesis. Indeed, vascular endothelial growth factor A (VEGFA) displayed a pregnancy-dependent up-regulation by 1.5-fold in the current study.

The expression of leukemia inhibitory factor (*LIF*), a member of the interleukin-6 (IL6) family of cytokines, was up-regulated at Day 22 and Day 28 of pregnancy. An increase in LIF expression at the conceptus–maternal interface during capsule attenuation has been reported previously [44]. Leukemia inhibitory factor binds to a heterodimeric receptor composed of LIFR and IL6ST. While interleukin 6 signal transducer (IL6ST) displayed a tendency towards higher expression at Day 28 of pregnancy, we observed a pregnancy-specific up-regulation of the corresponding leukemia inhibitory factor receptor (*LIFR*), underlining a role for LIF in equine pregnancy.

Inhibin beta C (*INHBC*) and inhibin beta E (*INHBE*) were expressed at higher levels at Day 14 than at Days 22 and 28 of pregnancy, while inhibin A (*INHA*) expression levels did not change with day of pregnancy. The expression of inhibin subunits by endometrial tissue has previously been described for women and mares [45, 46], and the expression of activin receptors at the uteroplacental interface in the mare has been reported [47]. Activin receptor expression levels did not vary with pregnancy stage in the current study. A lower expression of inhibin beta A has been reported for women suffering repeated miscarriages, yet a defined role for inhibin/activin in endometrial function remains to be determined.

A number of serpins, a superfamily of proteins with similar structures, were differentially regulated. *SERPINI1*, *SERPINA10*, *SEPRING1*, and *SERPINB5* were up-regulated with progression of pregnancy, while uterine serpin (*SERPINA14*) was not differentially regulated. *SEPRPINB9*, *SERPINA6*, and *SERPINB9* displayed lower expression in mares that failed to extend the luteal phase following the administration of oxytocin, a reflection of the progesterone-dependent nature of serpins.

The complement system was amongst the canonical pathways significantly enriched in transcripts differentially expressed between Day 14 and Day 22/28 of pregnancy. In a microarray analysis of bovine endometrial tissue collected at Day 17 from pregnant and cyclic dairy cattle, complement pathway genes were amongst the up-regulated genes [48]. In women, endometrial expression of the complement components C3 and DAF is modulated by hCG, likely involving a cross-talk with progesterone [49] and impaired expression of the complement system has been reported in women with infertility, underlining the importance of the complement system in establishment/maintenance of pregnancy [50–52]. Up-regulation of the complement system at the embryo-maternal interface has been speculated to protect the developing embryo against pathogens [48], a relevant aspect given the immune-suppressive nature of pregnancy. The expression of complement components 7 and 8 (*C7* and *C8*) was confirmed using in situ hybridization. Staining for *C7* was weaker than *C8*, correlating with the lower transcript abundance of *C7* than *C8* upon RNA sequencing analysis. Except for the C1 complex, the expression of the complement factors was low based on the RNA sequencing data, which is reflected in the staining pattern observed for *C7* and *C8* (scattered punctuated dots in glandular epithelial cells). Up-regulation of *C7* at Day 28 of pregnancy and up-regulation of *C8* at Day 14 of pregnancy seems to be mediated via their expression in luminal epithelial cells which, individually, displayed strong staining intensity for either *C7* or *C8*. As mentioned earlier, a number of serpins were up-regulated. SERPING1, also known as C1-inhibitor, is an inhibitor of the complement system [53] and we confirmed protein expression through immunohistochemistry. Protein expression was strongest in luminal epithelial cells and slightly less pronounced in glandular epithelial cells. Stromal cells displayed weak staining intensity. No obvious difference in staining intensity was visible between samples collected at Day 14 and Day 28 of pregnancy, which is not surprising given the limited suitability of IHC as quantitative tool. SERPING1 could serve as a checkpoint modulating unwanted complement mediated immune responses at the embryo-maternal interface. We have previously reported fibrinogen as a mediator of cell adhesion in equine pregnancy [54]. The protease plasmin is the primary fibrinolysin degrading fibrin and is generated from the zymogen plasminogen [55]. SERPING1 is an inhibitor of plasmin [56] and might therefore also play a role in maintaining the presence of fibrin at the embryo-maternal interface.

Results were furthermore compared to a meta-analysis which determined endometrial receptivity associated genes of human endometrium through the analysis of 76 samples from “pre-receptive” phase endometrium and 88 samples from “receptive” phase endometrium [35]. Of the 57 genes identified as markers of endometrial receptivity in this meta-analysis, 13 were differentially expressed in the present study and 30 were expressed but not differentially regulated. Beta-defensin 1 transcript was 13-times more abundant at Day 28 than Day 14. Defensins are small peptides that are components of the innate immune system and exert antimicrobial activity [57]. A previous study confirmed the expression of beta-defensin in equine endometrium and immunohistochemical analysis revealed strong protein expression in luminal epithelial cells and in superficial glandular epithelial cells [58]. Like the present study, the meta-analysis highlights the importance of the complement cascade pathway. Secreted phosphoprotein 1, *SSP1*, also known as osteopontin, was up-regulated in “receptive” phase endometrium. A role for osteopontin as an adhesion molecule for implantation during ovine and porcine pregnancy has been studied in detail [59]. In the present study, a ten-fold lower expression of *SPP1* was observed at Day 22 and Day 28 of pregnancy compared to Day 14 of pregnancy. We have previously reported a pronounced decrease of *SSP1* mRNA expression from Day 8 to Day 14 conceptuses and had hypothesized that the reduced expression of osteopontin contributes to the prolonged preimplantation phase of the equine conceptus [60]. Considering the observed down-regulation of osteopontin in the present study, it appears that osteopontin does not play a central role in conceptus-adhesion during the time of fixation during equine pregnancy. As mentioned earlier, we have previously reported fibrinogen as a mediator of cell adhesion in equine pregnancy and given the down-regulation of *SSP1* observed in the present study, fibrinogen gains in significance as a central molecule in conceptus adhesion during equine pregnancy.

## Conclusion

The present transcriptome study revealed limited changes in gene expression between Day 22 and Day 28 of pregnancy, while considerable changes in transcriptional activity occurred between the second and third week of pregnancy. Analysis of differential gene expression between the second and third week of pregnancy highlight the importance of complement factors cascade during early pregnancy. In follow-up studies the importance of the complement system in the context of early pregnancy loss should be evaluated.

## Supporting information

S1 Table Transcripts differentially expressed between samples collected at Day 14, Day 22, and Day 28 of pregnancy. (XLSX)

S2 Table TPM values, p-value and fold changes for all transcripts differentially expressed. (XLSX)

S3 Table Comparison of genes expressed in equine endometrium at Days 14, 22 and 28 of pregnancy to 57 genes identified in a meta-analysis of endometrial-receptivity associated genes of human endometrium. (XLSX)

S4 Table Primers utilized for real-time RT-PCR. (XLSX)

S1 Fig (JPG)
