# Supplementary figures and images for: Transcriptional profiling of equine endometrium before, during and after capsule disintegration during normal pregnancy and after oxytocin-induced luteostasis in non-pregnant mares
Source: PLoS One. 2021 Oct 6;16(10):e0257161. doi: 10.1371/journal.pone.0257161 (PMC8494348; doi:10.1371/journal.pone.0257161)

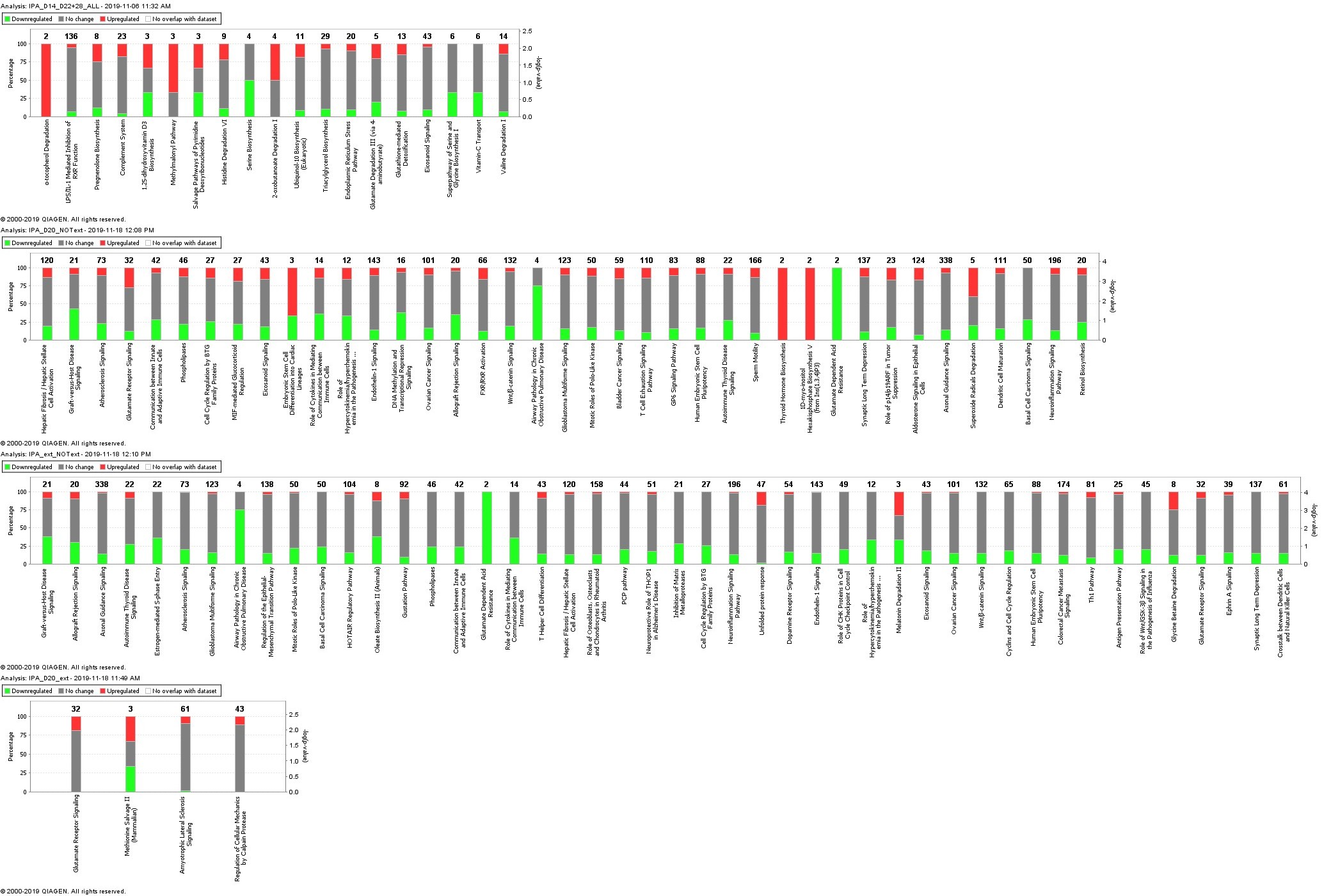

Supplement: S1 Fig — (JPG) [file pone.0257161.s005.jpg]
